# Divalent Metal Ion Differentially Regulates the Sequential Nicking Reactions of the GIY-YIG Homing Endonuclease I-BmoI

**DOI:** 10.1371/journal.pone.0023804

**Published:** 2011-08-22

**Authors:** Benjamin P. Kleinstiver, Wesley Bérubé-Janzen, Andrew D. Fernandes, David R. Edgell

**Affiliations:** 1 Department of Biochemistry, Schulich School of Medicine & Dentistry, The University of Western Ontario, London, Ontario, Canada; 2 Department of Applied Mathematics, The University of Western Ontario, London, Ontario, Canada; New England Biolabs Inc., United States of America

## Abstract

Homing endonucleases are site-specific DNA endonucleases that function as mobile genetic elements by introducing double-strand breaks or nicks at defined locations. Of the major families of homing endonucleases, the modular GIY-YIG endonucleases are least understood in terms of mechanism. The GIY-YIG homing endonuclease I-BmoI generates a double-strand break by sequential nicking reactions during which the single active site of the GIY-YIG nuclease domain must undergo a substantial reorganization. Here, we show that divalent metal ion plays a significant role in regulating the two independent nicking reactions by I-BmoI. Rate constant determination for each nicking reaction revealed that limiting divalent metal ion has a greater impact on the second strand than the first strand nicking reaction. We also show that substrate mutations within the I-BmoI cleavage site can modulate the first strand nicking reaction over a 314-fold range. Additionally, in-gel DNA footprinting with mutant substrates and modeling of an I-BmoI-substrate complex suggest that amino acid contacts to a critical GC-2 base pair are required to induce a bottom-strand distortion that likely directs conformational changes for reaction progress. Collectively, our data implies mechanistic roles for divalent metal ion and substrate bases, suggesting that divalent metal ion facilitates the re-positioning of the GIY-YIG nuclease domain between sequential nicking reactions.

## Introduction

Homing endonucleases are DNA endonucleases that primarily function as mobile genetic elements by introducing double-strand breaks or nicks at specific homing sites in naïve genomes [1], [2]. DNA repair, recombination and replication pathways repair the double-strand break (or nick) using the endonuclease-containing genome as a template, resulting in the mobilization of the homing endonuclease gene and surrounding DNA to the recipient genome [1]. In model laboratory systems, the efficiency of endonuclease-mediated homing is extraordinary, reaching 80–100% in some cases [3], [4], [5], implying that homing endonucleases can quickly spread through populations of naïve genomes. The rapid accumulation of genome sequence data has revealed an abundance of homing endonuclease genes in bacterial, archaeal, viral, and organellar genomes, including the mitochondrial and nuclear genomes of eukaryotes [6], [7]. Five distinct homing endonuclease families, defined by conserved amino acids, have been characterized to date. They are the LAGLIDADG, HNH, His-Cys box, GIY-YIG, and PD-(D/E)xK families [8], [9].

**Figure 1:**
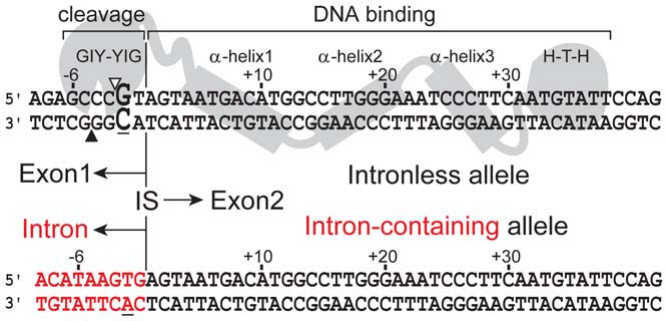
Model of I-BmoI interactions with intronless and intron-containing substrates based on DNA footprinting experiments. Shown is a schematic of the modular interaction of I-BmoI with the intronless *thyA* allele (upper), and the resultant changes to the target site upon intron insertion that generates the intron-containing allele (lower). Top- and bottom-strand nicking sites are indicated by open and filled triangles, respectively. The critical GC-2 base-pair is shown in enlarged bold-type font, and the intron insertion site (IS) is indicated by a vertical line with exon 2 downstream and either exon 1 or the intron (red) upstream.

Homing endonucleases have attracted much interest as potential reagents for manipulating complex genomes, and substantial effort has been devoted to reprogramming the specificity of a select few LAGLIDADG endonucleases to cleave clinically relevant targets [10], [11], [12], [13], [14]. Understanding the mechanism of DNA hydrolysis by homing endonucleases is critical for their utilization as reagents for targeted manipulation of complex genomes. From a mechanistic perspective, the least understood of the homing endonuclease families are the GIY-YIG enzymes. GIY-YIG homing endonucleases are modular in nature and contain an N-terminal cleavage domain of ∼100 amino acids that includes the class-defining GIY-YIG motif, and a C-terminal DNA-binding domain that is composed of distinct modules (Figure 1) [15], [16]. The N- and C-terminal domains are connected by a flexible linker that functions as a molecular ruler to position the catalytic domain at the correct distance on substrate from the DNA-binding domain [17], [18], [19], [20]. Two well-studied GIY-YIG endonucleases are the isocaudomers I-TevI and I-BmoI, encoded within group I introns interrupting the thymidylate synthase genes of bacteriophage T4 and Bacillus mojavensis, respectively [21], [22], [23]. I-TevI and I-BmoI introduce a staggered double-strand break by two independent and sequential nicking reactions at the same positions of their substrates, with the bottom (non-coding) strand of substrate nicked before the top (coding) strand [24], [25]. DNA bending assays and in-gel footprinting demonstrated that significant DNA distortions on the bottom strand occur independently of the first nicking reaction [24], [25]. Interestingly, studies with mutant DNA substrates revealed distinct sequence requirements for efficient double-strand break formation by each endonuclease. In particular, I-BmoI absolutely requires a GC base pair at position -2 immediately 3′ to the top-strand nicking site [26], yet displays no sequence preference for bases flanking the bottom-strand nicking site, while I-TevI requires an additional CG base pair 5′ to the bottom-strand nicking site for cleavage activity [27].

The GIY-YIG nuclease motif is not unique to homing endonucleases, as the domain is found in a variety of protein scaffolds [28], including DNA repair proteins [29], retrotransposable elements [30], and restriction endonucleases [31], [32], [33]. Structural studies of diverse GIY-YIG domains have revealed a compact α/β-fold composed of a central three-stranded antiparallel β-sheet flanked by three α-helices [29], [34], [35], [36], [37], [38]. A set of conserved residues comprise the single active site that uses a one-metal ion mechanism to catalyze DNA hydrolysis [36],[37]. The function of the GIY-YIG nuclease domain varies in a scaffold-dependent manner, as the domain has acquired additional structural units that are interspersed within the GIY-YIG domain to direct oligomerization or DNA binding. For instance, the GIY-YIG restriction enzyme Eco29kI utilizes additional folds to functions as a dimer [39], with each monomer nicking one strand of substrate. In the case of the UvrC nucleotide excision repair protein, the single GIY-YIG domain nicks 3′ to a lesion site to initiate a repair event [40]. The homing endonucleases are distinct from other GIY-YIG family members, as they have an extended structure and bind DNA as monomers [25]. They are believed to successively use the single active to generate a double-strand break [38], but how the active size is reorganized to accommodate different DNA strands for the sequential nicking reactions is unknown.

Here, we investigate the contributions of divalent metal ion and DNA sequence to the sequential nicking reactions of I-BmoI. We show that cleavage by I-BmoI is sensitive to the identity and concentration of divalent metal ion, and that the second strand nicking rate is affected to a greater extent by low metal ion concentrations than the rate of the first nicking reaction. DNA substrate mutations at key positions within the cleavage site of I-BmoI can differentially attenuate or completely abolish first- and second-strand nicking. Furthermore, we show that the GC-2 base pair is crucial for generating bottom strand minor-groove distortions that are a necessary prerequisite to cleavage.

## Results

### Magnesium is the preferred divalent metal ion for efficient and specific cleavage

**Figure 2:**
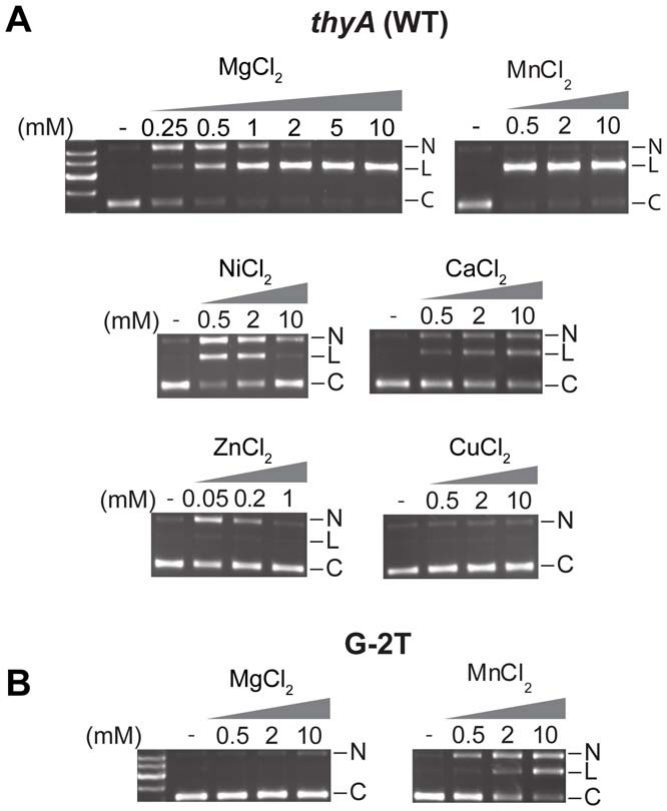
Magnesium is the preferred divalent metal ion for efficient and specific cleavage by I-BmoI. A. Representative gel images of time-point cleavage assays with I-BmoI performed on supercoiled substrate containing the intronless *thyA* target site. Reactions contained increasing concentrations of MgCl_2_, CaCl_2_, CuCl_2_, MnCl_2_, NiCl_2_, and ZnCl_2_. Lanes that lack I-BmoI (-) have 10 mM metal (1 mM for ZnCl_2_), and nicked (N), linear (L), and circular (C) plasmid forms are indicated to the right of each gel image. B. Representative gel images of I-BmoI cleavage assays performed on supercoiled substrate containing a mutation at the critical GC-2 basepair (G-2T). Reactions were performed in the presence of MgCl_2_ or MnCl_2_.

To determine the divalent metal ion preference for cleavage by I-BmoI, we tested various metals across a range of concentrations in assays with a supercoiled plasmid (pBmoHS) containing the I-BmoI intronless *thyA* target site (Figure 2). I-BmoI reaction progress can be visualized using supercoiled substrate, as the first nicking reaction generates a nicked plasmid intermediate and the second nicking reaction converts the nicked intermediate to linear product [41]. Cleavage assays were performed under single turnover conditions (protein excess) with a range of MgCl_2_, CaCl_2_, CuCl_2_, MnCl_2_, NiCl_2_, and ZnCl_2_ concentrations (Figure 2). Reactions with CoCl_2_ did not yield any products. The overall divalent metal ion preference of I-BmoI was Mn^2+^>Mg^2+^>Ni^2+^>Ca^2+^>Zn^2+^>> Cu^2+^ = Co^2+^. In particular, cleavage was extremely efficient in the presence of MnCl_2_ for all concentrations tested, while reactions in MgCl_2_ displayed a second strand defect at 0.25 mM MgCl_2_, near complete conversion of nicked intermediate to linear product at 2 mM MgCl_2_, and complete conversion to product at 10 mM MgCl_2_ (Figure 2A). NiCl_2_ appeared to be inhibitory at 10 mM, yet similar levels of cleavage were observed at 0.5 mM NiCl_2_ and MgCl_2_. Cleavage was observed in the presence of CaCl_2_ (albeit greatly reduced versus MgCl_2_), whereas ZnCl_2_ and CuCl_2_ were inhibitory at most concentrations tested, with the exception that 0.05 mM ZnCl_2_ supported nicking only. These findings correlate well with the typical roles ofF divalent metal observed for the majority of site-specific DNA endonucleases, including the tetrameric GIY-YIG restriction enzyme Cfr42I, apart from the observation that cleavage by Cfr42I was most efficient in CoCl_2_-containing buffers [31], [42], [43], [44].

Site-specific endonucleases have been shown to exhibit increased activity in the presence of manganese, but at the cost of fidelity [45], [46]. To determine if I-BmoI displayed a similar loss of fidelity in the presence of MnCl_2_ relative to MgCl_2_, we performed cleavage assays on a plasmid substrate with a G-2T mutation, against which I-BmoI is known to retain only limited activity (Figure 2B) [26]. We observed minimal nicking of the pBmoHS G-2T substrate by I-BmoI in the presence of 10 mM MgCl_2_, while 2 mM and 10 mM MnCl_2_ were condusive for cleavage (but reduced versus intronless *thyA* substrate). Collectively, these data suggested that the observed increase in efficiency in the presence of MnCl_2_ is partly due to decreased fidelity, allowing I-BmoI to cleave non-cognate sites. Furthermore, based on these results we determined that of the divalent metal ions tested, a selection of magnesium concentrations would provide the optimal level of efficiency and specificity.

### Limiting divalent metal ion has a more pronounced regulation of second strand nicking

**Figure 3:**
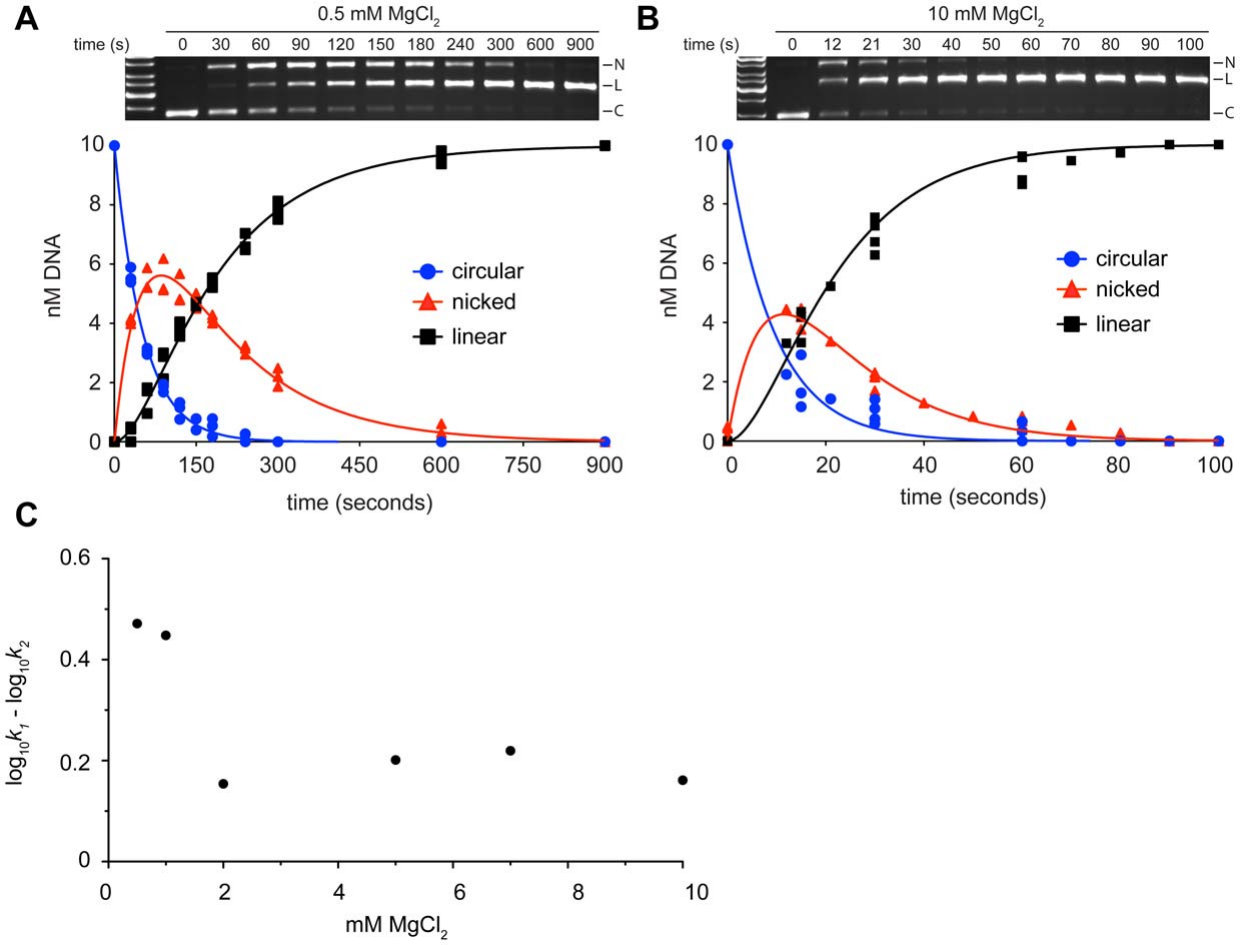
Limiting divalent metal ion has a greater effect on second strand than first strand nicking. Shown are representative images of time-course cleavage assays with supercoiled substrate containing the intronless *thyA* target site and I-BmoI in A. 0.5 mM MgCl_2_ and B. 10 mM MgCl_2_, as well as progress curves for each condition. Circular substrate (C), nicked intermediate (N) and linear product (L) are indicated on the gel images. Individual data points from three independent replicates are shown in the progress curves, and the solid continuous lines are the best fit of the data to equations 1 and 2. C. Plot of the log_10_ *k_1_* - log_10_ *k_2_* value for MgCl_2_ concentrations tested.

To gain insight into the substrate conversion process by I-BmoI, we selected a range of MgCl_2_ concentrations to dissect reaction progress as previous data indicated that the sequential reactions have distinct metal requirements [24]. We performed time-course cleavage assays with the *thyA* supercoiled substrate and determined rate constants based on the following reaction scheme:
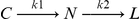
where k_1_ is the rate constant for conversion of supercoiled plasmid (C) to nicked intermediate (N), and k_2_ is the rate constant for conversion of nicked intermediate to linear product (L). Representative time course experiments in 0.5 mM and 10 mM MgCl_2_ are shown in Figure 3, with k_1_ and k_2_ rate constants for all MgCl_2_ concentrations tested summarized in Table 1. We observed a 5.2-fold reduction in k_1_ when MgCl_2_ was reduced from 10 mM to 0.5 mM MgCl_2_, while a 12.2-fold decrease was observed for k_2_. The distinct metal requirements for each nicking reaction is represented in the log_10_k_1_ - log_10_k_2_ versus MgCl_2_ concentration plot, which shows a more pronounced decrease in k_2_ as the concentration of MgCl_2_ is reduced (Figure 3C). These data suggest that divalent metal ion regulates the rate of both nicking reactions, but that the second strand nicking reaction has a more stringent requirement for divalent metal than the first nicking reaction.

**Table 1:** Rate constants for first- and second-strand nicking reactions in different MgCl_2_ concentrations.

[MgCl_2_] (mM)	*k_1_* (s^−1^)^a^	*k_2_* (s^−1^)
10	0.10±0.007	0.078±0.008
7	0.10±0.005	0.060±0.003
5	0.079±0.004	0.050±0.002
2	0.056±0.001	0.036±0.001
1	0.037±0.0006	0.013±0.0004
0.5	0.019±0.0002	0.0064±0.0002

a *k_1_* and *k_2_* rate constants are reported as the best fit value of three independent experiments with standard error.

### Assays with mutant substrates reveal three distinct cleavage phenotypes

To further probe the contribution of divalent metal ion and DNA sequence to the sequential nicking reactions by I-BmoI, we performed time-course cleavage assays with various supercoiled mutant substrates at 2 mM and 10 mM MgCl_2_. We took advantage of a series of previously constructed substrates [26], whereby positions -6 through -1 of intronless *thyA* substrate were individually and in combination changed to the corresponding intron-containing sequence (Figure 1 and Table 2), which I-BmoI does not cleave. End-point assays with these substrates highlighted the importance of the GC-2 base pair in the generation of a DSB by I-BmoI [26]. The previous assays, however, were incapable of distinguishing defects in each independent nicking reaction because they were performed on linearized plasmid substrates in 10 mM MgCl_2_.

**Table 2:** Summary of cleavage data for intronless and mutant substrates.

							Rate constants (s^−1^) a			
	Substrate						2mM MgCl_2_		10mM MgCl_2_	
	-6	-5	-4	-3	-2	-1	*k_1_* b	*k_2_* c	*k_1_*	*k_2_*
*(intronless)*	**G**	**C**	**C**	**C**	**G**	**T**	0.057	0.026	0.11	0.074
**Class I**			-4A				0.050	0.028	0.10	0.057
(like wild-type)	-6T						0.057	0.024	0.087	0.058
**Class II**						-1G	0.031	0.0095	0.065	0.025
(rescue)				-3G			0.010	0.0084	0.025	0.025
		-5A					0.0095	0.016	0.031	0.042
			-4A	-3G			0.0082	0.0091	0.024	0.022
		-5A	-4A				0.0044	0.015	0.014	0.043
	-6T	-5A					0.011	0.017	0.028	0.048
	-6T	-5A	-4A				0.0045	0.015	0.014	0.047
**Class III**					-2T		0.00027	*n.d.* d	0.00061	*n.d.*
(no rescue)					-2T	-1G	*n.d.*	*n.d.*	*n.d.*	*n.d.*
				-3C	-2T		*n.d.*	*n.d.*	*n.d.*	*n.d.*
			-4A		-2T		*n.d.*	*n.d.*	*n.d.*	*n.d.*
		-5A			-2T		*n.d.*	*n.d.*	*n.d.*	*n.d.*
		-5A		-3C			0.00037	0.00053	0.0013	0.014
				-3G	-2T	-1G	*n.d.*	*n.d.*	*n.d.*	*n.d.*
		-5A		-3G		-1G	0.00016	*n.d.*	0.00035	0.0026
			-4A	-3G	-2T		*n.d.*	*n.d.*	*n.d.*	*n.d.*
	-6T		-4A		-2T		*n.d.*	*n.d.*	*n.d.*	*n.d.*
		-5A	-4A	-3G			0.00020	0.0017	0.00052	0.0091
		-5A	-4A	-3G	-2T		*n.d.*	*n.d.*	*n.d.*	*n.d.*
*(intron-containing)*	**T**	**A**	**A**	**G**	**T**	**G**	*n.d.*	*n.d.*	*n.d.*	*n.d.*

a Rate constants were determined from three independent experimental trials for all substrates.

b *k*_1_, the rate constant for the first nicking reaction that generates nicked intermediate from circular substrate (expressed as the median of a 95% confidence interval, see Table S3).

c *k*_2_, the rate constant for the second nicking reaction that generates the linear product (expressed as the median of a 95% confidence interval, see Table S3).

d *n.d.* not determined.

**Figure 4:**
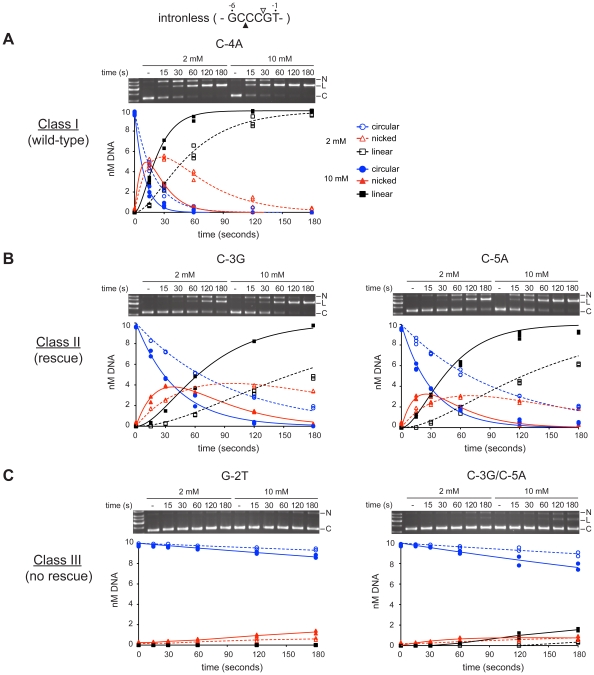
Magnesium concentrations reveal three distinct classes of cleavage site substitutions. Cleavage assays with I-BmoI were conducted on supercoiled plasmid substrates containing substitutions at positions -6 to -1 in the presence of 2 mM or 10 mM MgCl_2_ (see also Table 2). Mutant substrates were arranged into three classes. (A) Substrates that showed a phenotype similar to wild-type intronless *thyA* substrate (class I); (B) substrates that demonstrated poor cleavage in 2 mM MgCl_2_ and rescued cleavage in 10 mM MgCl_2_ (class II); (C) substrates with significantly reduced or no cleavage (class III). Shown are representative gel images of time-course cleavage assays, where the second lane from the left contains unreacted plasmid substrate (-). Nicked (N), linear (L), and circular (C) plasmid forms indicated to the right. Beneath each gel image is a graphical representation of reaction progress over time in 2 mM and 10 mM MgCl_2_ using dashed and solid lines, respectively. Data points representing three independent experiments for class I and II, and two experiments for class III are shown.

**Time-course assays using mutant cleavage site plasmids revealed that the substrates** segregated into three distinct classes (Figure 4 and Table 2). Class I mutants were defined as substrates that behaved essentially as intronless *thyA* substrate, showing slightly reduced nicking and slower conversion of nicked intermediate to linear product at 2 mM versus 10 mM MgCl_2_ (Figure 4A). The substrates were those with substitutions C-4A or G-6T, and the rate constants for the first and second strand nicking reactions in low and high MgCl_2_ were very similar to those obtained for intronless *thyA* substrate (Table 2 and Table S3).

Class II mutants were defined as substrates that exhibited a greater impairment of first and second strand nicking in low versus high MgCl_2_ conditions. This class included the singly mutated substrates T-1G, C-3G, and C-5A, as well as the multiply substituted C-3G/C-4A, C-4A/C-5A, C-5A/G-6T and C-4A/C-5A/G-6T substrates (Table 2). The general characteristics of substrates in this class were the accumulation and persistence of the nicked intermediate in 2 mM MgCl_2_ (which resulted in impaired double-strand break formation), and the rescue of cleavage in 10 mM MgCl_2_ resulting in the near complete conversion to linear product by 180 seconds (Figure 4B and Table 2). Rate constant analysis indicated a further segregation of substrates within class II by differential base-specific effects on first and second strand nicking reactions (Table 2). The T-1G substitution decreased the first (k_1_) and second (k_2_) strand rates by roughly 2- and 3-fold versus intronless *thyA* substrate in the presence of 2 mM and 10 mM MgCl_2_, respectively, suggesting a more profound modulation of second-strand nicking. The C-3G substitution, which displayed similar defects in the presence or absence of a C-4A substitution, generated approximately 5- and 3- fold reductions in k_1_ and k_2_ versus intronless substrate, regardless of MgCl_2_ concentration. Interestingly, the C-5A substitution, alone or in the context of a G-6T substitution, decreased k_1_ by roughly 6- and 3-fold in the presence of 2 mM and 10 mM MgCl_2_, respectively. The addition of a C-4A substitution to the C-5A template exacerbated the k_1_ defect to 12- and 9-fold in the presence of 2 mM and 10 mM MgCl_2_. Surprisingly, the C-5A substitution showed less of an influence on the second-strand nicking rate (k_2_), evidenced by a 1.5-fold rate reduction in both MgCl_2_ concentrations. This result was observed with the C-5A substitution alone or in the context of the double mutation with C-4A.

Class III mutants were defined as substrates with drastically reduced reaction rates that displayed little or no cleavage in either low- or high-metal ion conditions (Figure 4C and Table 2). All substrates with mutations at the -2 position fell into this category, in addition to substrates with mutations at both the -5 and -3 positions. Interestingly, we observed that the G-2T substitution alone, and in combination with substitutions at positions -1 or -4 and -6, demonstrated limited accumulation of nicked intermediate at 180s in high metal. First strand nicking rates (k_1_) for class III substrates were 84- to 314-fold reduced as compared to intronless *thyA* substrate, indicating a substantial first strand defect. Additionally, we observed that the substrates containing a combination of C-3G and C-5A mutations retained low levels of double-strand break formation after 180 s in 10 mM MgCl_2_, and that the addition of a T-1G or C-4A mutation did not abolish the low amount of cleavage. Importantly, the intron-containing substrate showed no evidence of nicking or cleavage after 180 seconds under either condition tested (Table 2).

Collectively, the cleavage assays indicated that substrates with individual mutations at positions -4 or -6 had little effect on first- or second-strand nicking in either low or high metal ion conditions. Additionally, substrates with single mutations at positions -1, -3 or -5 exhibited defects in both first- and second-strand nicking in low metal conditions and an observable rescue of cleavage by high divalent metal ion. Substrates with mutations at both the -3 and -5 positions were not significantly rescued by high metal ion, and those with mutations at position -2 were defective for cleavage under all conditions tested.

### In-gel footprinting reveals multiple minor groove distortions dependent on GC-2

The cleavage data suggested that divalent metal ion and the GC-2 base pair are required for efficient double-strand break formation. To gain further insight into the role of the GC-2 base pair in the cleavage pathway, we performed in-gel footprinting with the minor groove-specific reagent 1,10-copper phenanthroline (OP-Cu). We previously used in-gel footprinting to show that significant OP-Cu hypersensitive sites were localized to positions -2 and -1 of the bottom strand of the I-BmoI-*thyA* complex [24], consistent with protein-induced DNA distortions that make the minor groove more accessible to OP-Cu. The distortions were also present in I-BmoI-substrate complexes formed with the catalytic mutants R27A and E74A, implying that the distortions precede first-strand nicking and do not require the presence of a metal ion bound by E74 [24]. Previous footprinting studies, however, did not explore the requirement of the GC-2 base pair in formation of OP-Cu hypersensitive sites.

**Figure 5:**
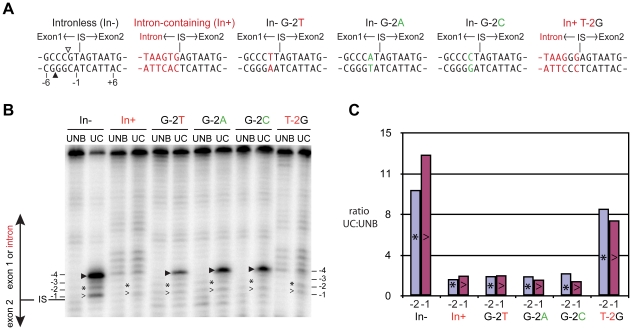
The GC-2 base pair is required for minor-groove distortion near the bottom-strand nick site. (A) Illustration of base pairs -6 to +7 of the intronless (In-), intron-containing (In+), and mutant 74-mer substrates used for in-gel 1,10-phenanthroline copper (OP-Cu) footprinting reactions. First- and second-strand nicking sites are indicated by open and filled triangles, respectively. (B) Representative denaturing gel image of OP-Cu footprinting reactions on bottom-strand labeled In-, In+, and mutant substrates. Nicked products at -4 and minor groove distortions at positions -2 and -1 are indicated using filled triangles, asterisks, and greater-than symbols, respectively. Sequence upstream of the intron insertion site (IS) varies whether the intronless or intron-containing substrate was used as a template. (C) Graphical representation of the minor groove sensitivity to OP-Cu at positions -2 (asterisk, blue) and -1 (greater than symbol, purple) for In-, In+, and mutant substrates, expressed as the ratio of normalized phosphorimager units of the upper complex (UC) and unbound substrate (UNB).

We performed in-gel footprinting with a 74-mer duplex oligonucleotide substrate corresponding to the intron-containing substrate (In+), which contains an alternative sequence upstream of the insertion site relative to the intronless substrate and notably has a G-2T substitution (Figure 5). As shown in Figures 5b and 5c, the OP-Cu hypersensitive sites at positions -2 and -1 on the bottom strand were significantly reduced in the UC consisting of I-BmoI-In+ substrate as compared to UC formed with intronless *thyA* substrate. We next mutated the GC-2 base pair within the context of the intronless substrate to TA-2, AT-2 or CG-2, and measured the extent of the OP-Cu hypersensitive sites at positions -2 and -1 on the bottom strand. These three mutations reduced the OP-Cu hypersensitive sites to the same extent as was observed with intron-containing substrate (Figure 5b and 5c), indicating the GC-2 base pair is critical for inducing DNA distortions that result in enhanced OP-Cu sensitivity. To provide further evidence for this model, we tested a T-2G substitution within the context of the intron-containing substrate (Figure 5a), reasoning that this mutation should restore the OP-Cu hypersensitivity if contacts to the GC-2 base pair are required for minor-groove distortions. Indeed, footprinting reactions on the T-2G intron-containing substrate revealed a restoration of OP-Cu hypersensitivity at positions -2 and -1 to approximately half of those observed on *thyA* intronless substrate. Products seen at the -4 position are indicative of bottom-strand nicks observed when I-BmoI-*thyA* complexes were formed in-gel in the absence of exogenously added metal [24], as Cu^2+^ is not a productive divalent metal ion for cleavage (Figure 2A).

### Modeling of an I-BmoI-substrate complex

**Figure 6:**
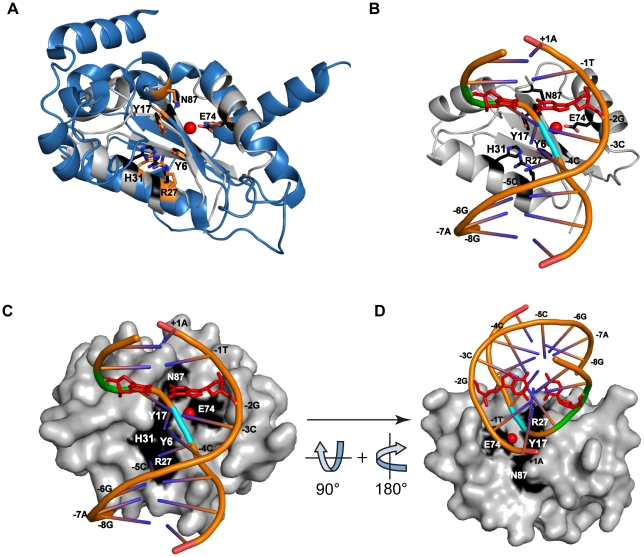
Model of I-BmoI GIY-YIG domain interactions with substrate. (A) Cartoon representation of the I-BmoI GIY-YIG domain homology model (gray) [41] aligned with the solution structure of related GIY-YIG restriction enzyme Eco29kI (3MX1, blue) [36]. The side chains of the conserved and catalytically relevant residues Y6, Y17, R27, H31, E74, and N87 of I-BmoI are shown in black, and equivalent Eco29kI residues in orange (Y49, Y76, R104, H108, E142, and N154). The position of divalent metal coordinated by Eco29kI is represented by the red sphere. (B) Cartoon representation of the I-BmoI homology model aligned with a segment of the DNA from the substrate-bound Eco29kI structure (3N1C,-4C to +5C). The top strand of the Eco29kI substrate is labeled according to the nucleotides of the I-BmoI intronless allele (-8G to +1A). The phosphate of the bottom strand nick site is highlighted in blue, the bottom strand distortions at positions -2 and -1 are shown in green, and the -2GC base pair is shown as a stick model in red. (C) Surface representation of the GIY-YIG domain for the model shown in panel (B). (D) Similar to panel (C), with a 90° rotation of the model on the horizontal axis, and a 180° rotation around the vertical axis.

To gain insight into the role of divalent metal ion and substrate contacts in the I-BmoI reaction pathway, we used the recently solved co-crystals of the GIY-GIY restriction enzymes Eco29kI and Hpy188I with their respective substrates to model an I-BmoI-substrate complex [36], [37]. Eco29kI and Hpy188I function as dimers to cleave palindromic sites, with each GIY-YIG monomer nicking one strand of the substrate[37], [39]. We first generated a homology model of residues 1-92 of I-BmoI based on the closely related I-TevI GIY-YIG domain structure [38]. Next, we superimposed the I-BmoI model on a monomer of the dimeric Eco29kI and Hpy188I structures by aligning residues 6-10 of I-BmoI with the structurally analogous amino acids of Eco29kI (residues 47-51) and Hyp188I (residues 61-65) (which included key structural resides of the β-sheet 1). We observed that the position of the I-BmoI active site residues (Y6, Y17, R27, H31, E74 and N87) aligned well with the homologous residues in both restriction enzymes (Figure 6A and data not shown), as was observed in two other structure superposition studies [36], [37]. To model the position of the substrate in the I-BmoI active site, we included the substrate DNA from the Eco29kI structure, and similar results were obtained using the DNA from the Hpy188I structure. As shown in Figure 6, the path of the DNA follows a previously hypothesized catalytic cleft that is lined by the active site residues of I-BmoI [41], with the metal ion coordinated by E74 positioned in close proximity to the bottom-strand scissile phosphate at position -4. Of particular interest are the OP-Cu hypersensitive sites at positions -2 and -1 of the bottom strand that result from a widening of the minor groove. These distortions could place the base edge of the bottom strand C of the critical GC-2 base pair within hydrogen bonding distance of a number of side chains, highlighting the importance of this base pair in the cleavage pathway. The model also reinforces the notion that the role of divalent metal ion in promoting second-strand nicking must be in repositioning of the substrate-DNA complex rather than by directly participating in DNA hydrolysis.

## Discussion

Enzymes that function as DNA endonucleases almost always require the presence of active site divalent metal ions [8], [42], [44]. In general, the preferred ion is magnesium, which acts to catalyze reaction progress by stabilizing negative phosphoanion transition states, or by acting as Lewis acids to modulate the pKa of coordinated water molecules [42], [44]. Metal-activated water molecules can function as catalytic agents by adopting the roles of nucleophiles and general bases, or by protonating leaving groups [44], [47]. Families of site-specific DNA endonucleases have distinct metal ion requirements, and generally function by either a one- or two-metal ion mechanism [44], [47]. The metal ion requirements for homing endonucleases are best understood for the LAGLIDADG family that have an active-site preference for magnesium, and can uniquely function by a two-metal mechanism where a third divalent metal ion is shared between two active sites [8], [48], [49]. The H-N-H homing endonuclease I-HmuI, which generates single-stranded nicks in DNA substrates, functions optimally at 1 mM MgCl_2_ or MnCl_2_ and nicks via a one-metal ion mechanism [43]. Studies of the related H-N-H colicin E9 revealed different divalent metal ion requirements for cleavage of distinct nucleic acid substrates, with magnesium promoting cleavage of dsDNA and zinc being more effective for nicking of ssDNA [50]. I-PpoI, a His-Cys box homing endonuclease, functions by a one-metal ion mechanism and requires zinc and magnesium for folding and cleavage, respectively [8], [51]. The focus of this paper, the GIY-YIG homing endonucleases, have traditionally been studied in the presence of magnesium that structural studies indicate is coordinated by a conserved active site glutamate (E74 in I-BmoI) [38]. GIY-YIG enzymes likely use a single-metal ion to promote DNA hydrolysis [36], [37], and our data agrees with studies of the tetrameric GIY-YIG restriction enzyme Cfr42I that revealed a broad tolerance to divalent-metal ion [31].

Among the GIY-YIG family of enzymes, there is tremendous diversity in how the GIY-YIG nuclease domain is utilized to hydrolyze DNA. For instance, the restriction enzymes Eco29kI and Hpy188I function as dimers [37], [39], with each GIY-YIG monomer nicking one strand, whereas the UvrC nucleotide excision repair protein uses a single GIY-YIG domain to nick 3′ to a mutagenic lesion [40]. In this respect, GIY-YIG homing endonucleases differ from characterized GIY-YIG enzymes that make a DSB in that a single catalytic domain is used in two ordered and sequential nicking reactions to generate a DSB [24], [25]. How the single active site of the GIY-YIG nuclease domain is reorganized on substrate between nicking reactions is unknown, but previous studies suggested an involvement of divalent metal ion in this process [24]. Our current study more accurately defines the roles of divalent metal ion and DNA substrate bases in this process as evidenced by reaction conditions that differentially affect k_1_ or k_2_. Not surprisingly, substrates that contained a mutation in the critical GC-2 base pair displayed drastically impaired rate constants, indicating a severe defect in first-strand nicking and an inability to form a DSB. Interestingly, bases surrounding the bottom-strand nicking site at positions -4 and -5 also resulted in reduced nicking rates when mutated. This observation was somewhat surprising as previous studies had not revealed a critical role of these positions for cleavage by I-BmoI [26], likely because past studies examined only linear product formation under high metal conditions rather than employing reactions conditions that could temporally dissect base-specific nicking effects. The I-BmoI-substrate model predicts that multiple amino acid side chains within the catalytic cleft project into the major groove near positions -4 and -5, implying that base pair substitutions result in altered contacts that consequently reduce k_1_. The observation that k_1_ defects associated with these substrates were significantly rescued in the presence of 10 mM MgCl_2_ suggests that magnesium can compensate for lost I-BmoI-substrate interactions by promoting alternative conformations of protein or substrate within the catalytic cleft that facilitate cleavage. Additionally, substitutions near the bottom strand-nicking site had a comparatively insignificant effect on the top strand-nicking rate (k_2_), which is in agreement with the bottom strand contacts being necessary only for k_1_.

A crucial aspect of the I-BmoI cleavage pathway is the introduction of a significant minor groove distortion in *thyA* substrate [24]; similar DNA-bending was also observed in I-TevI-td substrate complexes [25]. These protein-dependent DNA distortions are observed in the absence of exogenous magnesium, and the precise role of these distortions in the cleavage pathway is unknown. Parallels can be drawn with other site-specific endonucleases, as large DNA distortions have been shown to bring opposite strand scissile phosphates into close proximity [52]. Conversely, the natural curvature of nucleic acids is highly sequence dependent and can be influenced by the presence of divalent metal ions [53], [54], [55]. Interestingly, magnesium ions have been shown to induce much greater changes in the curvature of GC- versus AT-rich DNA [53]. In this respect, it is noteworthy that the DNA distortions introduced by I-BmoI and I-TevI occur in stretches of GC-rich DNA [24], [25], suggestive of distinct metal dependent and protein induced distortions that promote substrate conformational changes between nicking reactions. It is possible that aside from directly participating in catalysis, magnesium may induced a secondary substrate conformation that is distinct from that due to contacts to GC-2 base pair. Reduced rate constants observed for I-BmoI on mutant substrates can therefore be rationalized by lack of base-specific contacts to the GC-2 pair and to other positions, as well as the sequence-dependence of DNA distortions induced by magnesium. Decreased fidelity of I-BmoI on the G-2T substrate in the presence of manganese can alternatively be explained by different protein-DNA conformations promoted by manganese ions relative to magnesium, as they have differential effects on the natural curvature of DNA [56].

Similar roles for metal ions in promoting protein-DNA interactions and conformational changes required for efficient catalysis have been dissected in other enzyme systems where a single active site is used sequentially to perform multiple reactions [57]. Notably, sub-optimal magnesium concentrations can uncouple excision and strand transfer events, and manganese can reduce target site specificity of the Tn10 transposase [57]. However, the modular structure of I-BmoI and other GIY-YIG homing endonucleases resembles that of Type IIs restriction enzymes, including the well-characterized FokI that is a monomer in solution but transiently dimerizes to generate a DSB [58], [59]. The FokI cleavage mechanism has been suggested as a solution to how the single active site of GIY-YIG homing endonucleases is used to affect a double-strand break [38]. Interestingly, divalent metal ion has been shown to stabilize the FokI dimer/substrate complex [59], and it is possible that a reduction in the k_2_ rate constant we observe in low magnesium conditions reflects a defect in transient dimerization between I-BmoI monomers. No obvious dimer interface was evident in the structure of the I-TevI catalytic domain [38], which shares high identity with that of I-BmoI, but it is possible that dimerization determinants lie outside of the catalytic domain. Interestingly, the type IIs enzyme Eco31I also has a modular structure but binds and cleaves DNA as a monomer [60], suggestive of mechanistic similarities to I-BmoI and I-TevI.

## Materials and Methods

### Bacterial strains and plasmids

*E.coli* strains DH5α and ER2566 (New England Biolabs) were used for plasmid manipulations and protein expression, respectively. pTYBmoI was used to over-express a wild-type, codon-optimized version of I-BmoI for purification as previously described [41]. pBmoHS is a pBS derivative that contains an insert corresponding to 49 bp of the intronless *thyA* substrate [26]. A complete description of all plasmids used in this study is found in Table S1.

### Oligonucleotides

Oligonucleotides used in this study can be found in Table S2.

### Cleavage assays on plasmid substrates

Single time-point cleavage assays to examine metal dependence were performed in 10-µl volumes containing 20 mM Tris-HCl pH 8.0, 250 mM NaCl, 2.5% glycerol, 10 nM wild-type or G-2T pBmoHS, and various concentrations of MgCl_2_, CaCl_2_, CuCl_2_, MnCl_2_, NiCl_2_, and ZnCl_2_. Reactions were started by the addition of I-BmoI to final a concentration of 175 nM, allowed to proceed for 90 seconds at 37°C, and stopped by the addition of 4 µl stop dye (100 mM EDTA, 25% glycerol, 0.2% bromophenol blue). Stopped reactions were heated for 5 minutes at 95°C, cooled on ice for 5 minutes, electrophoresed on a 1% agarose gel, and stained in a 1xTAE solution containing ethidium bromide (Caledon) prior to analysis on an AlphaImager™3400 (Alpha Innotech). Time course cleavage reactions to determine Mg^2+^ dependence were performed on supercoiled pBmoHS in 115-µl volumes with conditions similar to those listed above, with either 0.5 mM, 1 mM, 2 mM, 5 mM, 7 mM, or 10 mM MgCl_2_. A 10-µl aliquot was removed prior to the addition of wild-type I-BmoI to final a concentration of 175 nM, and subsequent 10-µl aliquots were removed at ten time points into 4 µl stop dye. Stopped reactions were visualized as indicated above. Reaction progress was determined by the relative amount of circular substrate, nicked intermediate, and linear product. At least 3 independent trials were conducted for each MgCl_2_ concentration. Time course cleavage reactions with mutant pBmoHS substrates were performed in 65-µl volumes with conditions similar to those listed above, with either 2 mM or 10 mM MgCl_2_. A 10-µl aliquot was removed prior to the addition of wild-type I-BmoI to final a concentration of 175 nM, and subsequent 10-µl aliquots were removed at 15, 30, 60, 120, and 180 seconds into 4 µl stop dye. Mutant substrates were classified as follows: class I substrates displayed a reaction progress similar to wild-type intronless substrate, class II substrates displayed defects for both bottom and top-strand nicking reactions at 2 mM MgCl_2_ and ‘rescue’ of cleavage to near WT levels after 180 seconds in 10 mM MgCl_2_, and class III substrates showed severe to complete defects in 2 mM MgCl_2_ and no appreciable rescue of activity in 10 mM MgCl_2_ after 180 seconds. Cleavage assays for class I, II, and a selection of class III substrates (G-2T, C-3G/C-5A, T-1G/C-3G/C-5A, C-3G/C-4A/C-5A, and In+) were conducted as described above. For the remainder of class III substrates, only the accumulation of nicked intermediate or linear product above background levels was measured after a 180 second incubation with I-BmoI in 2 mM or 10 mM MgCl_2_. Three independent reactions were performed for substrates that gave measurable rate constants, and two independent trials were performed for class III substrates that displayed minimal or no cleavage. Rate constants for the reaction
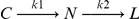
were estimated for the two-constant irreversible kinetic model using Prism5 (GraphPad Software) or via the Bayesian bootstrap [61]. The time-course data were fit to the three following equations 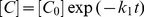
(1)

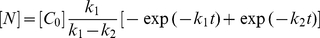
(2)

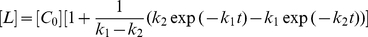
(3)where C_0_ is the initial concentration of circular substrate (in nM), [*N*] is the concentration of nicked DNA (in nM), [*L*] is the concentration of linear product (in nM), *k*_*1*_ is the first nicking rate constant (in s^−1^), *k* *_2_* is the second nicking constant (in s^−1^), and *t* is time (in seconds). For each bootstrap replicate, parameters were optimized for minimal discrepancy with the data under the half-taxi metric [62]. This procedure was found to be more robust than a standard least-squares estimation due to the constraint that the total amount of circular, nicked, and linear DNA is constant. Posterior parameter medians and 95% confidence intervals are reported in Table S3. The ratio of *k*_*1*_/*k* *_2_* at various MgCl_2_ concentrations was reported as the value of the log10*k*_*1*_ – log10*k* *_2_* to minimize over-weighting the end points if plotted on a linear scale.

### OP-Cu in-gel footprinting

1,10-phenanthroline copper (OP-Cu) footprinting experiments were conducted as previously described [24]. Substrates used included a 74-mer duplex oligonucleotide substrate corresponding to the intronless *thyA* target site (DE-116/DE-117), the intronless 74-mer with G-2T (DE-446/DE-447), G-2A (DE-459/DE-460), or G-2C (DE-461/DE-462) substitutions, the intron-containing 74-mer (DE-444/DE-445), and the intron-containing 74-mer with A-2G substitution (DE-463/DE-464) (see Table S2). Gel images were analyzed using ImageQuant 5.2 (GE Healthcare Life Sciences). To quantify hypersensitive sites observed in the OP-Cu footprint, two bands outside of the I-BmoI protection region were selected to normalize phosphorimager units (positions +9 and +10 for intronless substrates, and +11 and +12 for intron-containing substrates, relative to the intron insertion site). Hypersensitivity to OP-Cu at positions -1 and -2 was calculated by expressing the ratio of normalized phosphorimager units at sites in the shifted footprint (UC, upper complex) to the units in the unbound substrate reaction (UNB, unbound) [24].

### Molecular modeling

The I-BmoI GIY-YIG domain homology model was built as previously described [41], and alignments to annotated structures were performed using MacPyMOL v1.2r. Residues 6-10 of the homology model were aligned to residues 47-51 of the solution structure of E142Q Eco29kI (3MX1) (Figure 6A) [36]. A subsequent alignment was performed with the I-BmoI homology model and a single subunit of the Y49F/L69K Eco29kI structure in complex with its 18 base pair substrate (3NIC) [36]. For illustration purposes, only nucleotides (-4)-CCCGCGGGC-(+5) of the Eco29kI substrate were shown. Additional alignments were performed using structures of the I-TevI GIY-YIG domain (1KM0 and 1LN0) in place of the I-BmoI homology model [38], and of Hpy188I in complex with substrate (3OQG) in place of the Eco29kI structure [37], yielding nearly identical results.

## Supporting Information

Table S1 **Strains and plasmids used in this study.**
(DOC)

Table S2 **Oligonucleotides used in this study.**
(DOC)

Table S3 **Confidence intervals for rate constants determined on mutant substrates in low and high MgCl2 concentrations.**
(DOC)
