# Supplementary material for: Divalent Metal Ion Differentially Regulates the Sequential Nicking Reactions of the GIY-YIG Homing Endonuclease I-BmoI
Source: PLoS One. 2011 Aug 22;6(8):e23804. doi: 10.1371/journal.pone.0023804 (PMC3161791; doi:10.1371/journal.pone.0023804)
Supplement: Table S1 — Strains and plasmids used in this study. (DOC) [file pone.0023804.s001.doc]

**Supplemental Table 1**. Strains and plasmids used in this study.

|  | Description | Source |
| --- | --- | --- |
| **Strains** |  |  |
| DH5α | F^-^, φ80d*lacZ*ΔM15, Δ(*lacZYA-argF*)U169, *deoR*, *recA*1, *endA*1, *hsdR*17(rk^-^, mk^+^), *phoA*, *supE*44, λ^-^, *thi*-1, *gyrA*96, *relA*1 | Invitrogen |
| ER2566 | F- λ- fhuA2 [lon] ompT lacZ::T7 gene 1 gal sulA11 Δ(mcrC-mrr)114::IS10 R(mcr-73::miniTn10-TetS)2 R(zgb-210::Tn10)(TetS) endA1 [dcm] | N.E.B. |
| **Plasmids** |  |  |
| pTYBmoI | pTYB1 derivative containing the 266 amino acid codon-optimized I-BmoI gene | Ref 1. |
| pBmoHS | pBS derivative containing a 48-base pair XbaI/BamHI insert corresponding to the intronless *B. mojavensis thyA* gene | Ref 2. |
| pDE212 | Similar to pBmoHS, with the intron-containing sequence upstream of the intron insertion site (substitutions relevant to this study are: T-1G, G-2T, C-3G, C-4A, C-5A, and G-6T) | Ref 2. |
| pDE213 | Similar to pBmoHS, with a T-1G substitution | Ref 2. |
| pDE214 | Similar to pBmoHS, with a G-2T substitution | Ref 2. |
| pDE215 | Similar to pBmoHS, with a C-3G substitution | Ref 2. |
| pDE216 | Similar to pBmoHS, with a C-4A substitution | Ref 2. |
| pDE217 | Similar to pBmoHS, with a C-5A substitution | Ref 2. |
| pDE218 | Similar to pBmoHS, with a G-6T substitution | Ref 2. |
| pDE219 | Similar to pBmoHS, with T-1G and G-2T substitutions | Ref 2. |
| pDE220 | Similar to pBmoHS, with G-2T and C-3G substitutions | Ref 2. |
| pDE221 | Similar to pBmoHS, with G-2T and C-4A substitutions | Ref 2. |
| pDE222 | Similar to pBmoHS, with G-2T and C-5A substitutions | Ref 2. |
| pDE223 | Similar to pBmoHS, with C-3G and C-4A substitutions | Ref 2. |
| pDE224 | Similar to pBmoHS, with C-3G and C-5A substitutions | Ref 2. |
| pDE225 | Similar to pBmoHS, with C-4A and C-5A substitutions | Ref 2. |
| pDE227 | Similar to pBmoHS, with T-1G, G-2T, and C-3G substitutions | Ref 2. |
| pDE228 | Similar to pBmoHS, with T-1G, C-3G, and C-5A substitutions | Ref 2. |
| pDE229 | Similar to pBmoHS, with G-2T, C-3G, and C-4A substitutions | Ref 2. |
| pDE230 | Similar to pBmoHS, with G-2T, C-4A, and G-6T substitutions | Ref 2. |
| pDE231 | Similar to pBmoHS, with C-3G, C-4A, and C-5A substitutions | Ref 2. |
| pDE232 | Similar to pBmoHS, with C-4A, C-5A, and G-6T substitutions | Ref 2. |
| pDE233 | Similar to pBmoHS, with G-2T, C-3G, C-4A, and C-5A substitutions | Ref 2. |

1. Kleinstiver, B.P., Fernandes, A.D., Gloor, G.B. and Edgell, D.R. (2010) A unified genetic, computational and experimental framework identifies functionally relevant residues of the homing endonuclease I-BmoI. *Nucleic Acids Res*, **38**, 2411-2427.
2. Edgell, D.R., Stanger, M.J. and Belfort, M. (2003) Importance of a single base pair for discrimination between intron-containing and intronless alleles by endonuclease I-*Bmo*I. *Curr Biol*, **13**, 973-978.
