# Supplementary material for: Divalent Metal Ion Differentially Regulates the Sequential Nicking Reactions of the GIY-YIG Homing Endonuclease I-BmoI
Source: PLoS One. 2011 Aug 22;6(8):e23804. doi: 10.1371/journal.pone.0023804 (PMC3161791; doi:10.1371/journal.pone.0023804)
Supplement: Table S2 — Oligonucleotides used in this study. (DOC) [file pone.0023804.s002.doc]

**Supplemental Table 2:** Oligonucleotides used in this study

| **Name** | **Sequence (5'-3')** | **Notes** |
| --- | --- | --- |
| DE-37 | CCACCTTGAGGTAAGAGCCCGTAGTAATGACATGGCCTTGGGAAATCCCTTCAATGTATTCCAG | Top strand intronless *thyA* 64mer from +22 to -42 relative to the intron insertion site |
| DE-38 | CTGGAATACATTGAAGGGATTTCCCAAGGCCATGTCATTACTACGGGCTCTTACCTCAAGGTGG | Bottom strand intronless *thyA* 64mer from +22 to -42 relative to the intron insertion site |
| DE-116 | AAACTCCACCTTGAGGTAAGAGCCCGTAGTAATGACATGGCCTTGGGAAATCCCTTCAATGTATTCCAGTACAA | Top strand intronless *thyA* 74mer from +27 to -47 relative to the intron insertion site |
| DE-117 | TTGTACTGGAATACATTGAAGGGATTTCCCAAGGCCATGTCATTACTACGGGCTCTTACCTCAAGGTGGAGTTT | Bottom strand intronless *thyA* 74mer from +27 to -47 relative to the intron insertion site |
| DE-444 | AGTAGCGACTTCTACTGAACATAAGTGAGTAATGACATGGCCTTGGGAAATCCCTTCAATGTATTCCAGTACAA | Top strand intron-containing *thyA* 74mer from +27 to -47 relative to the intron insertion site |
| DE-445 | TTGTACTGGAATACATTGAAGGGATTTCCCAAGGCCATGTCATTACTCACTTATGTTCAGTAGAAGTCGCTACT | Bottom strand intron-containing *thyA* 74mer from +27 to -47 relative to the intron insertion site |
| DE-446 | AAACTCCACCTTGAGGTAAGAGCCCTTAGTAATGACATGGCCTTGGGAAATCCCTTCAATGTATTCCAGTACAA | Top strand intronless *thyA* 74mer from +27 to -47 relative to the intron insertion site, with a G-2T substitution |
| DE-447 | TTGTACTGGAATACATTGAAGGGATTTCCCAAGGCCATGTCATTACTAAGGGCTCTTACCTCAAGGTGGAGTTT | Bottom strand intronless *thyA* 74mer from +27 to -47 relative to the intron insertion site, with a C-2A substitution |
| DE-459 | AAACTCCACCTTGAGGTAAGAGCCCATAGTAATGACATGGCCTTGGGAAATCCCTTCAATGTATTCCAGTACAA | Top strand intronless *thyA* 74mer from +27 to -47 relative to the intron insertion site, with a G-2A substitution |
| DE-460 | TTGTACTGGAATACATTGAAGGGATTTCCCAAGGCCATGTCATTACTATGGGCTCTTACCTCAAGGTGGAGTTT | Bottom strand intronless *thyA* 74mer from +27 to -47 relative to the intron insertion site, with a C-2T substitution |
| DE-461 | AAACTCCACCTTGAGGTAAGAGCCCCTAGTAATGACATGGCCTTGGGAAATCCCTTCAATGTATTCCAGTACAA | Top strand intronless *thyA* 74mer from +27 to -47 relative to the intron insertion site, with a G-2C substitution |
| DE-462 | TTGTACTGGAATACATTGAAGGGATTTCCCAAGGCCATGTCATTACTAGGGGCTCTTACCTCAAGGTGGAGTTT | Bottom strand intronless *thyA* 74mer from +27 to -47 relative to the intron insertion site, with a C-2G substitution |
| DE-463 | AGTAGCGACTTCTACTGAACATAAGGGAGTAATGACATGGCCTTGGGAAATCCCTTCAATGTATTCCAGTACAA | Top strand intron-containing *thyA* 74mer from +27 to -47 relative to the insertion site, with a A-2G substitution |
| DE-464 | TTGTACTGGAATACATTGAAGGGATTTCCCAAGGCCATGTCATTACTCCCTTATGTTCAGTAGAAGTCGCTACT | Bottom strand intron-containing *thyA* 74mer from +27 to -47 relative to the insertion site, with a T-2C substitution |
