# Supplementary material for: Divalent Metal Ion Differentially Regulates the Sequential Nicking Reactions of the GIY-YIG Homing Endonuclease I-BmoI
Source: PLoS One. 2011 Aug 22;6(8):e23804. doi: 10.1371/journal.pone.0023804 (PMC3161791; doi:10.1371/journal.pone.0023804)
Supplement: Table S3 — Confidence intervals for rate constants determined on mutant substrates in low and high MgCl2 concentrations. (DOC) [file pone.0023804.s003.doc]

**Supplemental Table 3.** Confidence intervals for rate constants determined on mutant substrates in low and high MgCl_2_ cocentrations.

|  |  |  |  |  |  |  |  | 2mM MgCl_2_ | | | | | | |  | 10mM MgCl_2_ | | | | | | |
| --- | --- | --- | --- | --- | --- | --- | --- | --- | --- | --- | --- | --- | --- | --- | --- | --- | --- | --- | --- | --- | --- | --- |
|  | Substrate | | | | | |  | *k*_1_ (s^-1^) ^a^ | | |  | *k*_2_ (s^-1^) ^b^ | | |  | *k*_1_ (s^-1^) | | |  | *k*_2_ (s^-1^) | | |
|  | -6 | -5 | -4 | -3 | -2 | -1 |  | low median high | | |  | low median high | | |  | low median high | | |  | low median high | | |
| intronless | **G** | **C** | **C** | **C** | **G** | **T** |  | 0.0531 | 0.0574 | 0.0628 |  | 0.0236 | 0.0260 | 0.0281 |  | 0.0762 | 0.1110 | 0.1310 |  | 0.0680 | 0.0738 | 0.0759 |
| **Class I** |  |  | -4 |  |  |  |  | 0.0461 | 0.0501 | 0.0645 |  | 0.0207 | 0.0279 | 0.0561 |  | 0.0922 | 0.1044 | 0.1178 |  | 0.0537 | 0.0571 | 0.0591 |
| *(like wild-type)* | -6 |  |  |  |  |  |  | 0.0504 | 0.0572 | 0.0610 |  | 0.0239 | 0.0241 | 0.0244 |  | 0.0763 | 0.0872 | 0.0937 |  | 0.0556 | 0.0575 | 0.0586 |
| **Class II** |  |  |  |  |  | -1 |  | 0.0266 | 0.0308 | 0.0315 |  | 0.0095 | 0.0095 | 0.0101 |  | 0.0534 | 0.0651 | 0.0755 |  | 0.0245 | 0.0245 | 0.0255 |
| *(rescue)* |  |  |  | -3 |  |  |  | 0.0100 | 0.0102 | 0.0104 |  | 0.0083 | 0.0084 | 0.0085 |  | 0.0243 | 0.0246 | 0.0263 |  | 0.0247 | 0.0249 | 0.0257 |
|  |  | -5 |  |  |  |  |  | 0.0095 | 0.0095 | 0.0095 |  | 0.0150 | 0.0155 | 0.0156 |  | 0.0282 | 0.0309 | 0.0312 |  | 0.0409 | 0.0421 | 0.0428 |
|  |  |  | -4 | -3 |  |  |  | 0.0080 | 0.0082 | 0.0086 |  | 0.0088 | 0.0091 | 0.0094 |  | 0.0243 | 0.0244 | 0.0245 |  | 0.0218 | 0.0221 | 0.0225 |
|  |  | -5 | -4 |  |  |  |  | 0.0042 | 0.0044 | 0.0050 |  | 0.0150 | 0.0153 | 0.0156 |  | 0.0128 | 0.0136 | 0.0143 |  | 0.0411 | 0.0434 | 0.0448 |
|  | -6 | -5 |  |  |  |  |  | 0.0103 | 0.0105 | 0.0106 |  | 0.0164 | 0.0170 | 0.0190 |  | 0.0269 | 0.0277 | 0.0287 |  | 0.0467 | 0.0484 | 0.0502 |
|  | -6 | -5 | -4 |  |  |  |  | 0.0043 | 0.0045 | 0.0046 |  | 0.0146 | 0.0154 | 0.0155 |  | 0.0129 | 0.0135 | 0.0143 |  | 0.0453 | 0.0468 | 0.0486 |
| **Class III** |  |  |  |  | -2 |  |  | 0.00025 | 0.00027 | 0.00028 |  | *n.d.* | *n.d.* | *n.d.* |  | 0.00059 | 0.00061 | 0.00063 |  | *n.d.* | *n.d.* | *n.d.* |
| *(no rescue)* |  |  |  |  | -2 | -1 |  | *n.d.* | *n.d.* | *n.d.* |  | *n.d.* | *n.d.* | *n.d.* |  | *n.d.* | *n.d.* | *n.d.* |  | *n.d.* | *n.d.* | *n.d.* |
|  |  |  |  | -3 | -2 |  |  | *n.d.* | *n.d.* | *n.d.* |  | *n.d.* | *n.d.* | *n.d.* |  | *n.d.* | *n.d.* | *n.d.* |  | *n.d.* | *n.d.* | *n.d.* |
|  |  |  | -4 |  | -2 |  |  | *n.d.* | *n.d.* | *n.d.* |  | *n.d.* | *n.d.* | *n.d.* |  | *n.d.* | *n.d.* | *n.d.* |  | *n.d.* | *n.d.* | *n.d.* |
|  |  | -5 |  |  | -2 |  |  | *n.d.* | *n.d.* | *n.d.* |  | *n.d.* | *n.d.* | *n.d.* |  | *n.d.* | *n.d.* | *n.d.* |  | *n.d.* | *n.d.* | *n.d.* |
|  |  | -5 |  | -3 |  |  |  | 0.00035 | 0.00037 | 0.00040 |  | 0.00026 | 0.00053 | 0.00150 |  | 0.00085 | 0.00130 | 0.00145 |  | 0.0000 | 0.0138 | 0.0148 |
|  |  |  |  | -3 | -2 | -1 |  | *n.d.* | *n.d.* | *n.d.* |  | *n.d.* | *n.d.* | *n.d.* |  | *n.d.* | *n.d.* | *n.d.* |  | *n.d.* | *n.d.* | *n.d.* |
|  |  | -5 |  | -3 |  | -1 |  | 0.00008 | 0.00016 | 0.00025 |  | *n.d.* | *n.d.* | *n.d.* |  | 0.00032 | 0.00035 | 0.00036 |  | 0.0022 | 0.0026 | 0.0028 |
|  |  |  | -4 | -3 | -2 |  |  | *n.d.* | *n.d.* | *n.d.* |  | *n.d.* | *n.d.* | *n.d.* |  | *n.d.* | *n.d.* | *n.d.* |  | *n.d.* | *n.d.* | *n.d.* |
|  | -6 |  | -4 |  | -2 |  |  | *n.d.* | *n.d.* | *n.d.* |  | *n.d.* | *n.d.* | *n.d.* |  | *n.d.* | *n.d.* | *n.d.* |  | *n.d.* | *n.d.* | *n.d.* |
|  |  | -5 | -4 | -3 |  |  |  | 0.00018 | 0.00020 | 0.00021 |  | 0.00008 | 0.00169 | 0.00271 |  | 0.00039 | 0.00052 | 0.00070 |  | 0.0087 | 0.0091 | 0.0106 |
|  |  | -5 | -4 | -3 | -2 |  |  | *n.d.* | *n.d.* | *n.d.* |  | *n.d.* | *n.d.* | *n.d.* |  | *n.d.* | *n.d.* | *n.d.* |  | *n.d.* | *n.d.* | *n.d.* |
| intron-containing | **T** | **A** | **A** | **G** | **T** | **G** |  | *n.d.* | *n.d.* | *n.d.* |  | *n.d.* | *n.d.* | *n.d.* |  | *n.d.* | *n.d.* | *n.d.* |  | *n.d.* | *n.d.* | *n.d.* |

^a^ *k*_1_, the mean of the 95% confidence interval for the rate constant that describes the first nicking reaction, which generates nicked intermediate from circular substrate

^b^ *k*_2_, the mean of the 95% confidence interval for the rate constant that describes the second nicking reaction, which generates linear product

*n.d.*, not determined
